# Comparison of filter-based and software-based image enhancement systems for endoscopic swallowing diagnostics: a head-to-head study

**DOI:** 10.1007/s00405-025-09413-w

**Published:** 2025-05-05

**Authors:** Almut C. Niessen, Julia Glinzer, Jana Zang, Christina Pflug

**Affiliations:** 1 Center for Clinical Neurosciences, Department of Voice, Speech and Hearing Disorders, University Medical Center Hamburg-Eppendorf, University Dysphagia Center, Martinistr. 52, 20246 Hamburg, Germany; 2 University of Lübeck, Institute for Health Sciences, Ratzeburger Allee 160, 23562 Lübeck, Germany

**Keywords:** Image enhancement in dysphagia diagnostics, Deglutition, Direct diagnostics of microaspirations, Dysphagia diagnostics

## Abstract

**Purpose:**

Flexible endoscopic evaluation of swallowing (FEES) is a gold standard for diagnosing swallowing disorders. This study investigates two image enhancement technologies usable in FEES—hardware filtering (Narrow Band Imaging, NBI^®^) and software filtering (Professional Image Enhancement Technology, PIET^®^)—to assess their effectiveness in improving the visibility of food dyes.

**Methods:**

This Head-to-head study compared NBI^®^ and PIET^®^ by creating 190 videos showcasing food dyes diluted from 1:10 to 1:100,000, tested in the oral cavities of four volunteers. Four raters evaluated the maximum visible dilution for both systems across all colors. Sixteen snippets representing eight colors (red, yellow, blue, green, purple, orange, black, and white) at a 1:10 dilution were analyzed by 14 raters, including two with no prior FEES experience, all blinded to the filtering method.

**Results:**

The study used a point system to assess subjective image quality, color intensity, and contrast to the mucosa. Both systems produced similar hues for yellow and red and their secondary colors. PIET was preferred for red and purple, while NBI was favored for yellow, green, and orange. For blue, black, and white (all showing no intensification), PIET was nearly unanimously chosen. Raters agreed 100% on the maximum visible dilution, showing no significant difference between systems; both enhanced visibility tenfold.

**Conclusions:**

Both image enhancement systems improved the visibility of specific food dyes effectively. Each method has distinct advantages. The choice between them depends on personal preference and available systems.

**Supplementary Information:**

The online version contains supplementary material available at 10.1007/s00405-025-09413-w.

## Introduction

Objective swallowing diagnostics are crucial for identifying and treating swallowing disorders, thereby reducing serious health complications [1, 2]. Alongside videofluoroscopic swallowing study (VFSS), flexible endoscopic evaluation of swallowing (FEES) is the gold standard in swallowing diagnostics [3]. During FEES, the swallowing process is examined transnasally by endoscopy. While VFFS allows observation of the intradeglutitive process, FEES is limited to pre- and post-deglutition, as a “white-out” effect obscures the endoscopic view during swallowing. Although FEES demonstrates greater sensitivity in detecting aspiration [4], small amounts of aspirated material may go unnoticed during the white-out phase.

Recently, several studies have explored the potential use of image enhancement to detect small amounts of aspirate [5–8]. The new filter-based technology, also called “high sensitivity FEES” or “FEES+”, has been developed to extend the capabilities of FEES, allowing for the detection of even the smallest amounts of aspirated material by amplifying images up to tenfold [9]. Using less food dye in the bolus offers several advantages: It minimizes staining of the oral mucosa [10], the risk of hyperactivity in children is reduced [11], and the risk by an aspirated bolus is diminished [12]. Enhanced visibility is particularly crucial, as it enables examiners to detect micro-aspirations immediately during the examination.

The significance of micro-aspirations in swallowing diagnostics has been widely discussed in current literature [13–15]. Authors have explored the potential link between micro-aspirations and aspiration pneumonia. However, the current literature does not demonstrate direct visibility of micro-aspirations or discuss the relevance of the amount of aspirated bolus. Instead, in the publications mentioned above, the presence of higher levels of Pepsin A or alpha-amylase in the subglottic area or new radiologic findings in the lung are regarded as indirect evidence of micro-aspirations. In contrast, FEES can directly observe aspirations; FEES + has the capability to detect even very small quantities of aspirate, making it the first method available to visualize micro-aspirations directly.

Conventional FEES employs white light (WL), which encompasses the full spectrum of colors visible to the human eye. WL consists of electromagnetic waves of varying wavelengths with visible light ranging from approximately 380 nm (violet) to about 750 nm (red). When light interacts with an object, it absorbs certain wavelengths and reflects others. The human eye then processes these reflected wavelengths. For example, a red object appears red because it absorbs all wavelengths except red. If only green light is directed at the red object, it will absorb all the wavelengths and appear black. This property can be effectively used in image enhancement.

There are two types of systems for enhancing endoscopic imaging: filter-based systems like NBI^®^ (Narrow Band Imaging) and software-based systems like PIET^®^ (Professional Image Enhancement Technology). NBI uses mechanical filters to enhance images by sending only blue and green light onto a surface. PIET achieves enhancement in the same spectrum using software filters. Both filtering methods must be applied during the examination, as existing WL images cannot be enhanced afterward. Software filters for image enhancement are widely available and commonly used in everyday camera systems [16].

Both NBI and PIET were initially developed for tumor diagnostics [17–19]. Neither company is - understandably - forthcoming about the background of its filtering methods. In tumor diagnostics, it is important to emphasize the area around the two main absorption peaks of hemoglobin: around 415 nm in the yellow range (the Soret band) and around 540 nm in the red spectrum. Hemoglobin absorbs blue light at around 415 nm, making blood vessels and flow in the superficial mucosal layer stand out [20]. As a result, the contrast between blood vessels and surrounding tissues is enhanced, with blood vessels that absorb more blue light appearing darker and the surrounding tissue appearing lighter due to reflecting more blue light. The use of NBI in swallowing diagnostics enhances specific yellow and red color tones and their combinations [9]. Selecting a suitable food dye for boluses is essential to achieve optimal enhancement through NBI, as only certain colors are optimally highlighted. The best colors for enhancement in endoscopic swallowing examinations using software-based systems such as PIET have yet to be determined.

Own previous tests indicated that applesauce or bread that has been baked with green color is correspondingly better visible with NBI. However, we found that typical water-soluble food dyes do not stain milk effectively. It is important to note that this study focused solely on comparing a water solution to evaluate the effectiveness of a software-based system (PIET) with filter-based image enhancement (NBI) for swallowing diagnostics. The main objective was to assess whether both enhancement methods are equally effective in improving images for diagnosing swallowing issues. The study also aimed to determine whether the two systems enhance different colors.

## Methods

### Devices and software used

The NBI examinations were performed using a flexible high-definition rhino-laryngo-videoscope containing an integrated switchable NBI filter and a xenon light source (ENF-VH, 3.9 mm, Olympus Medical Systems Corp.). Endoscopic videos were recorded using the rpSzene system v.6 (Rehder).

The PIET examinations were performed using the Xion Spectar Video Nasopharyngoscope XN HD 3.6 mm, equipped with integrated LED lighting and a microphone in the handle on the EndoSTROBE System. The videos were recorded using the DiVAS software on the MATRIX DS data station with the EndoSTROBE EL SPECTAR – Processor.

While NBI uses mechanical filters that are visibly—and audibly—‘clicking in’, PIET has four software modes for enhancing tumor visibility: *Chromo*, *Lumino*, a combination of both, and *Spectro*.

All types of data were tabulated in Excel (2016, Microsoft) and exported for further analysis to SPSS (Version 27, IBM). A descriptive data analysis was conducted.

### Implementation

The study framework design was oriented on a previous study that focused on NBI [9]. From this study, it is known that the oral cavity can serve as an easily accessible substitute for the larynx, but the dilutions through saliva in the larynx are about 1:10 higher than in the oral cavity. Initially, images using NBI and PIET were captured of test tubes to compare various food colors at different dilutions (1:10, 1:100, 1:1000, 1:10 000, 1:20 000, and 1:100 000). The dilution was in tap water, and the colors were all water-soluble. In each examination, 1 ml of fluid was administered via an insulin syringe.

Following this, images were taken in the oral cavity of three female volunteers, age 30, 40, and 56. All volunteers were well hydrated, fed, and free of oral issues, including oral discoloration, which is common at 20.8% [21]. The examination was performed starting with the highest dilution and ending with 1:10. Both devices were used immediately following each other for each dilution, and the order was switched for each subsequent examination; for example, 1:10 000 in one color was first examined with NBI, then with PIET, the following 1:1000 in the same color was then examined first with PIET, then with NBI.

In PIET, the starting mode was set to “Spectro” since selecting the preferred mode directly was impossible. The PIET standard was filmed following this, as Spectro is the last of the device’s four modes (see also online resource Figure S1). For NBI, the starting mode was random, as there is only one possible switch. The examination was conducted with the volunteer’s mouth open, using an endoscope positioned about 1 cm outside the gum line between the teeth to achieve an optimal view of the tongue. A comparison of the examination results for both open- and closed-mouth scenarios can be found in the supplementary materials.

Preliminary tests showed that residual color staining of the tongue lasted for hours and affected the examination of subsequent colors. Therefore, several test subjects were necessary for the study to be conducted in a reasonable amount of time. An additional problem is visible in the comparisons of oral cavity and larynx in the healthy volunteer in the supplemental photos (figures S2 and S3): A healthy volunteer usually is not able to produce large amounts of residues or even an aspiration [3]. In the previous study, the oral cavity was proven to be a valid representation of the larynx with NBI. It was demonstrated that saliva dilution renders filter-sensitive colors in the larynx ineffective at concentrations below 1:1000, while 1:10 000 is still visible in the oral cavity [9].

This study also examined a fourth healthy volunteer to test PIET in the larynx (see online resource Figures S2 and S3). All healthy volunteers gave written informed consent, and the study was conducted in conformity with the Helsinki Declaration [20]. The main focus of the examinations was on the images from the oral cavity, resulting in 190 videos. From these videos, films representative of the eight main colors (yellow, red, blue, green, purple, orange, white, and black) were chosen, all at a dilution of 1:10 (closest to the dilution used in FEES), finally resulting in 16 videos (eight with NBI, eight with PIET) for further evaluation. From these videos, snippets were cut to focus on the relevant areas for comparing the two methods. Relevant areas showed the filtered colors without the telltale switching of filters (visible change under NBI, written name of the used filter in the films with PIET), attempting to blind the raters to the used method. Neither the volunteers nor the raters received any stipends.

### Rating

The 16 videos were presented without sound to 14 persons individually on a tablet (Huawei Media Pad M5 lite 10) in a randomized order for rating. The tablet was used to avoid the raters being influenced by possible quality differences between the screens of the two devices. All individuals explicitly confirmed they were not color-blind before rating. The raters included physicians, speech-language pathologists (SLP), nurses, and an engineer, all with different amounts of FEES experience, and additionally, two individuals with no FEES experience (a physician and a student). These two were included to assess the possible bias of the other raters, who had extensive experience with NBI but none with PIET. The raters were asked to estimate their experience with FEES, NBI, and PIET on three individual visual analog scales (VAS).

PIET and NBI recordings for each color were presented next to each other; the raters could watch the clips as often as they wanted. The raters were asked whether the image quality differed between both systems. In addition to the overall impression, they were asked to assess the color intensity and contrast to the mucosa.

To obtain a uniform score for each color, the preferred method received one point, and both methods received one point if the score was ’equal‘. The points from all raters were added for each color and method, resulting in a sum score of a maximum of 14 and a minimum of 0 points (see Table 1).

To determine the maximum possible dilution, four individuals (two physicians and two SLPs) also compared the maximum dilution for visibility of PIET and NBI for each color to determine if both methods reached similar results with image enhancement. The maximum dilution of 1:100 000 in a test tube was also compared with water without dye.

## Results

The median professional experience of the 14 raters was 16 years (IQR 24). The median experience (including the two raters without experience) with FEES was 7.1 (IQR 3,42), with NBI in FEES 6.8 (IQR 2,28), and with PIET in FEES 0 (IQR 0,85), rated on a 10 cm VAS. Without the two raters without experience, the median values of the VAS were FEES 7.45, NBI in FEES 7.0, and PIET in FEES 0. The raw data can be found in the online resources (Table S1).

**Fig. 1:**
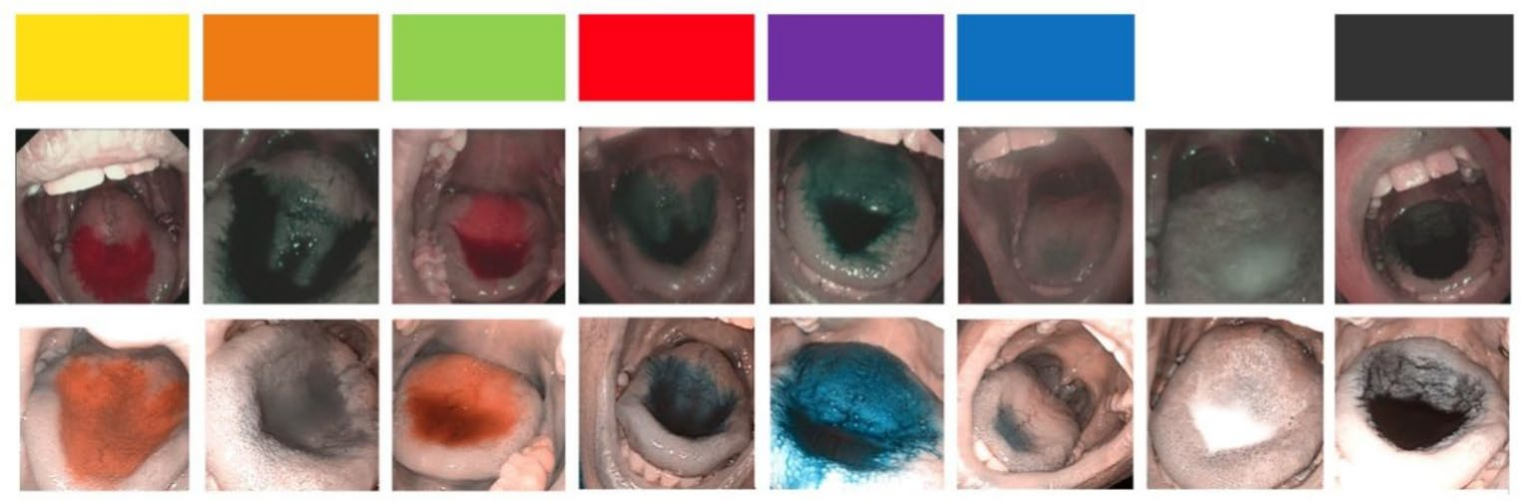
Comparison between NBI (above) and PIET (bottom) – all pictures with a 1:10 dilution. On the very top, the original colors. Note the difference in brightness between NBI and PIET

All colors that changed significantly under NBI in the oral cavity also changed to similar hues under PIET. Both methods change yellow and its secondary color green to red for NBI or orange for PIET (see Fig. 1). While red and its secondary color purple change to turquoise under NBI, they change to blue under PIET (see Fig. 1). Orange - a mixture of both changing colors - changes to grey/black. For a summary of the changes in appearance, see Fig. 1. An overview of the colors tested can be found in the online resources (Table S2).

**Fig. 2:**
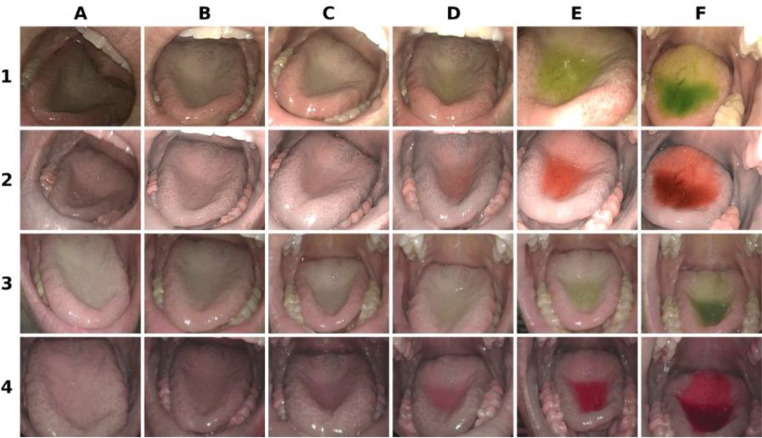
Dilution of ‘light green’ - top row software filtered white-light, row 2 PIET Spectro, row 3 mechanically filtered white-light, row 4 NBI. From left to right: 1:100 000 (**A**), 1:20 000 (**B**), 1:10 000(**C**), 1:1 000 (**D**), 1:100 (**E**), 1:10 (**F**)

Regarding the maximum possible dilution, both methods achieve the same enhancement and can be diluted similarly. All four voters agreed 100% on this. An example of the dilutions can be found in Fig. 2. The raters agreed that 1:20 000 appeared similar to water under both WL and enhancement, while 1:10 000 shows a light color tinge, both with NBI and PIET when seen in a video, while the color is not seen using WL.

In rating the oral cavity videos, most raters favored PIET for the colors red (PIET 40 points, NBI 12 points) and purple (PIET 38 points, NBI 26 points). NBI was the preferred choice for yellow (NBI 38 points, PIET 24 points) and its secondary color, green (NBI 32 points, PIET 21 points). This was also the case for orange (NBI 37 points, PIET 10 points). For blue (PIET 42 points, NBI 2 points), black (PIET 37 points, NBI 9 points), and white (PIET 41 points, NBI 3 points), all raters almost uniformly favored PIET. For more details about the rating, see Table 1 and Table S1 in the online resources.

**Table 1:**
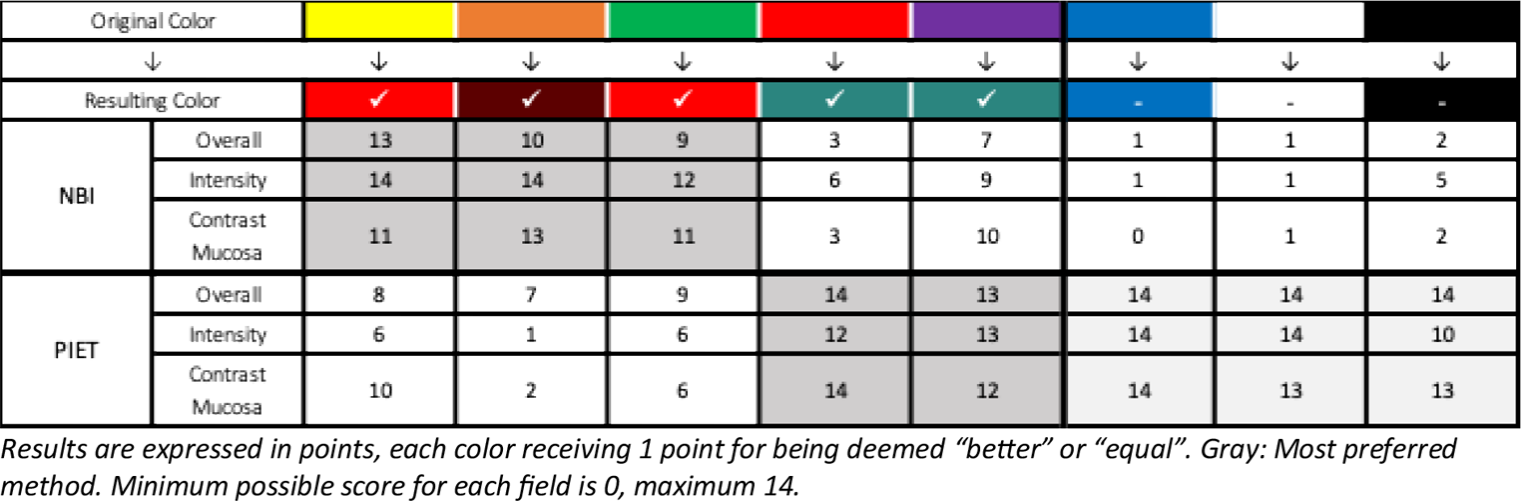
Detailed assessment of the individual focal areas surveyed in the PIET/NBI comparison

### Secondary findings

**Fig. 3:**
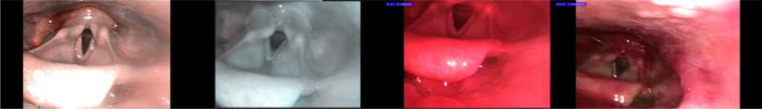
From left to right: PIET Spectro with small amounts of green food dye filtered to orange, second picture: PIET spectro after one swallow of red food dye, third picture: Change back to WL after trying all PIET modes and last picture after one swallow under standard WL without switching again

During a FEES procedure on a healthy volunteer with PIET, the software enhancement seemed to encounter difficulties when larger amounts of red food dye were used (Fig. 3). After the administration of green food dye (see Fig. 3 left image), small residues in the right piriform sinus remained, which appeared orange with PIET. Subsequently, a bolus (one teaspoon of puree – IDDSI 4) with red food dye 1:10 was given while PIET remained switched on (Fig. 3, second image from left). This caused significant fading and distortion of the color, which persisted for several seconds while switching through the four other modes - even under WL (see Fig. 3, third image), without the volunteer swallowing. Only after the volunteer swallowed again did the image normalize under WL (Fig. 3, far right). A video of the situation can be found in the online resources.

## Discussion

Both techniques, NBI (filter-based) and PIET (software-based), can be used to enhance colored boluses in the oral cavity tenfold. While NBI only uses one filter, PIET offers four filter modes for tumor diagnostics. However, among these modes, only *Spectro* appears to be suitable for swallowing diagnostics, as the other modes do not alter or improve the visibility of food dyes. For a demonstration, see online resource Figure S1 in the supplemental materials.

**Fig. 4:**
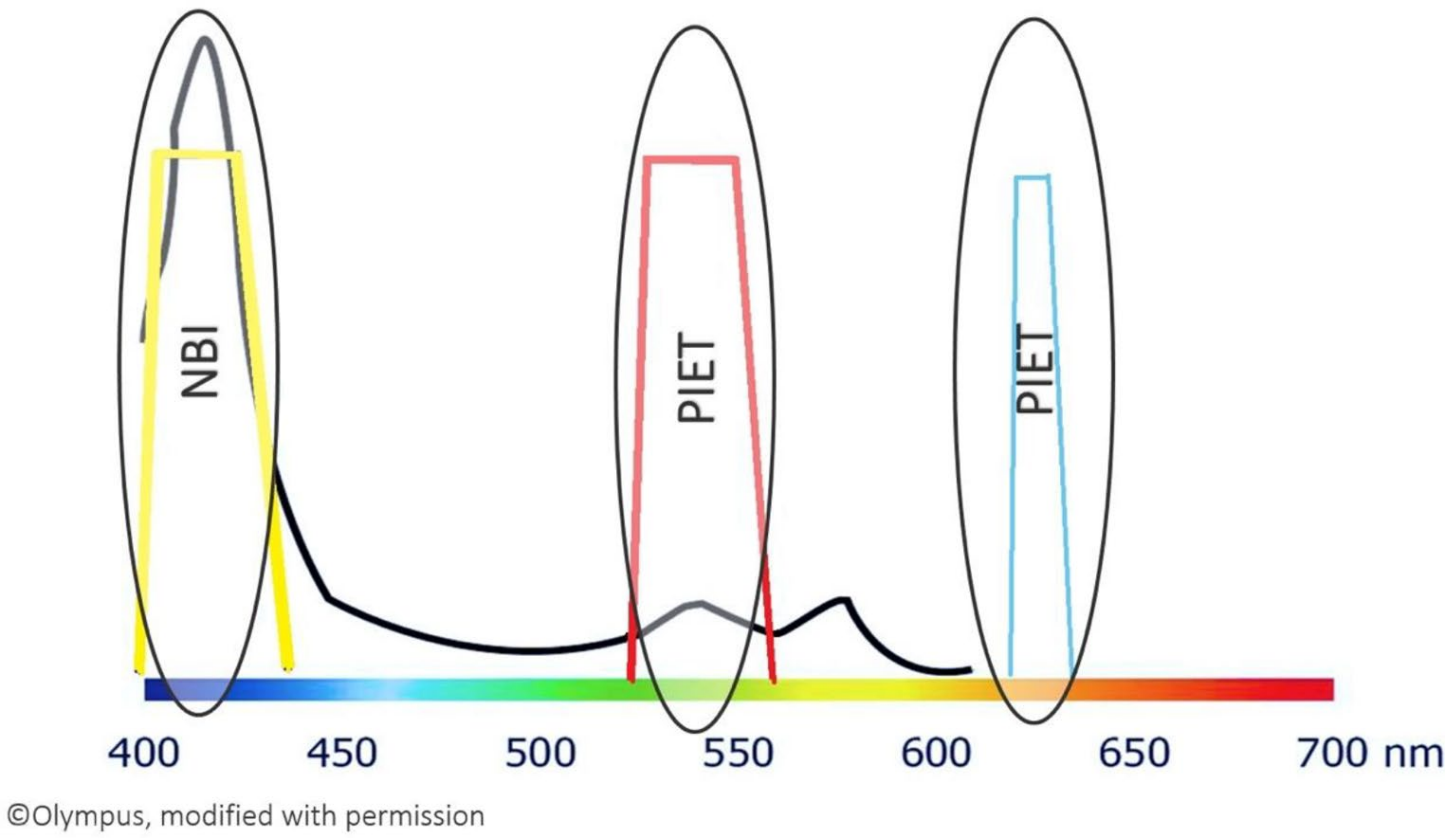
Absorption spectrum of hemoglobin (black line) and the NBI and PIET filters (yellow and red trapezoid). The x-axis represents the color spectrum and corresponding wavelengths. The more prominent peak in the blue spectrum is preferred for NBI, the smaller peak in the green spectrum is preferred for PIET. The trapezoids are in the color visible to the human eye if this wavelength is absorbed and only the color is reflected (yellow in the blue spectrum, red in the green spectrum, blue in the orange spectrum). Black and white are not considered colors in this context; white comprises all wavelengths, and black is the absence of all colors. Both can, therefore, not be placed in this diagram. Blue is not on the curve of hemoglobin and, thus, not enhanced/changed

The raters in this study preferred PIET for red and purple, while NBI was regarded better for yellow, green, and orange. No distinguishing factor could be pointed at in the present data that led to the preference of either technique. An explanation could be the better visibility of PIET when filtered – the hemoglobin peak in the green spectrum (increasing red) is much smaller than the peak in the blue spectrum (increasing yellow). This is visualized and explained in Fig. 4.

The filter-based images produced by NBI systems appear darker than white-light images [22]. Software-enhanced images (such as PIET) do not alter the brightness as much, as seen in Fig. 1, or are even enhanced in brightness, as visible in Fig. 2. NBI light is blue—visible in the online resource Figure S4—while PIET light stays white.

During the rating of the overall impression, several raters commented that the filtered PIET results were more pleasing because they were brighter. This was remarked on several times, regardless of individual experience with FEES and image enhancement. Despite this, the NBI-filtered results were preferred for yellow, orange, and green - even by the raters without experience with FEES. With the large (hemoglobin-)peak for yellow and its secondary colors, the darkening seems less relevant, and the mechanical filter can demonstrate its strengths. The darkening interferes with the small peak of red and its secondary colors, and the brighter amplification with software is perceived as better. This has also been shown in simulations in NBI – the superficial vessel contrast is better for the 415 nm (blue spectrum) peak than for the 540 nm (green spectrum) peak [20].

The difference in brightness probably also leads to PIET being preferred in non-amplified colors (blue, black, white). However, due to the lack of enhancement, these colors are unsuitable for clinical use with amplification and need a higher concentration of color [9].

Mechanical filters are more stable and do not show software errors, which are, of course, only possible with virtual amplification, as can be seen in Fig. 3. “Overexposure” can occur here. An objective measure of intensity for these types of images might be obtained by analyzing them using techniques such as ImageJ reprocessing or colorimetry.

This can only be corrected with a loss of time, in which the patients may swallow or clear their pharynx. In fact, as can be seen in the supplemental film of this “overexposure”, the picture only normalized after the volunteer swallowed. As a consequence, transient aspiration may go without notice. However, this problem only occurred once and was not observed in the oral cavity.

## Limitations

The VAS comparison of the raters’ NBI and PIET experience shows that the group’s expertise with the PIET device was limited. In contrast, the group had several years of experience with NBI. Consequently, the software filtering endoscope was considerably newer than the mechanical filtering endoscope, which might also influence the rating of the images’ quality.

We found that on the device we used for comparison with NBI, we had to cycle through all the PIET modes to get to the mode we wanted. Since Spectro is the last mode in the series, we could only directly compare WL and image intensification by first setting Spectro, checking the view, and then switching to WL. With NBI, we typically start with WL and then switch to filtered light to detect aspirations that are not visible with WL. This is not a problem when comparing colors in the oral cavity, but it could be a limitation when examining patients. After contacting the company directly, we were informed that changing the modes’ order is currently impossible. A future software update might address this, but it is not yet in sight.

The open mouth and visible teeth might cause a different white balance in the teeth in the endoscopy compared to the pharynx; this might influence the results in the pharynx when using software filters. However, it does not influence the workings of the hardware filter [9].

## Conclusion

Both image enhancement systems amplify specific food dyes tenfold. Each method has its strengths and weaknesses. Mechanical filters darken the image, especially affecting the weaker filtered color red and its secondary colors. Software filters may produce flawed images that can only be corrected with a loss of time. Ultimately, the choice between the two methods comes down to personal preference and depends on the examiner’s individual taste.

## Electronic supplementary material

Below is the link to the electronic supplementary material.

Supplementary Material 1

Supplementary Material 2

Supplementary Material 3
