# Supplementary material for: Comparison of filter-based and software-based image enhancement systems for endoscopic swallowing diagnostics: a head-to-head study
Source: Eur Arch Otorhinolaryngol. 2025 May 5;282(7):3739–45. doi: 10.1007/s00405-025-09413-w (PMC12321645; doi:10.1007/s00405-025-09413-w)
Supplement: Supplementary file 1 — Supplementary Material 1 [file 405_2025_9413_MOESM1_ESM.docx]

**Supplemental Figures and Tables**

Figure S1 Comparison of PIET filters – top in a healthy volunteer, larynx without food dye, bottom “light green” 1:100 in the oral cavity. From left to right: PIET standard – lumino – chromo – lumino/chromo and spectro. The name of the filter is printed in blue on the screen.


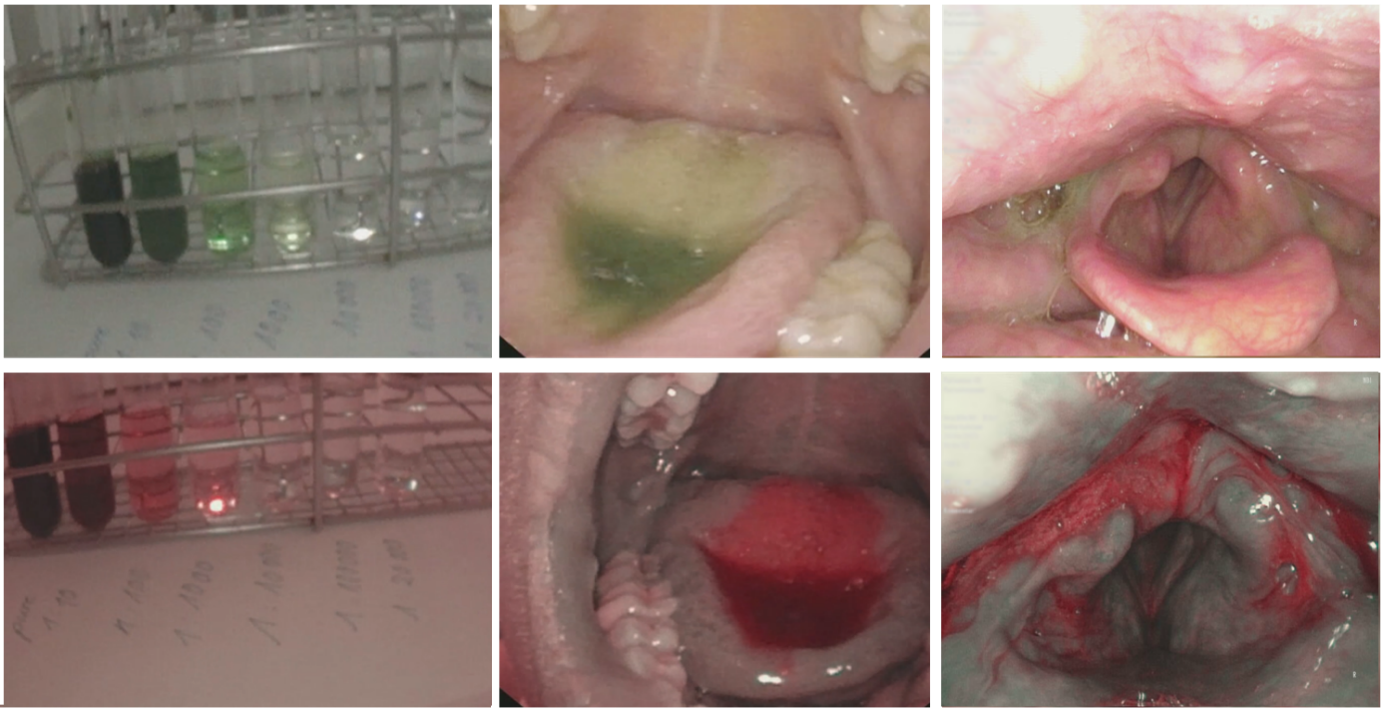


Figure S2 Comparison between white-light with Olympus endoscope (top) and light filtered with NBI; left test tubes, middle oral cavity, right larynx of a patient with micro-aspiration, for demonstration purposes and not part of the study (patient images courtesy of University Dysphagia Center).


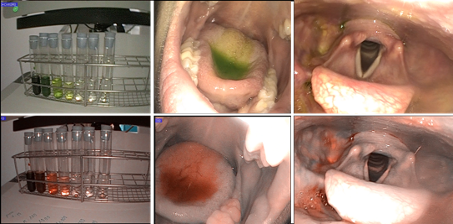


Figure S3 Comparison between white-light with Xion endoscope (top) and filtered with PIET Spectro. From left to right: Test tubes, oral cavity, larynx. Laryngeal examination in a healthy volunteer (images courtesy of the author).


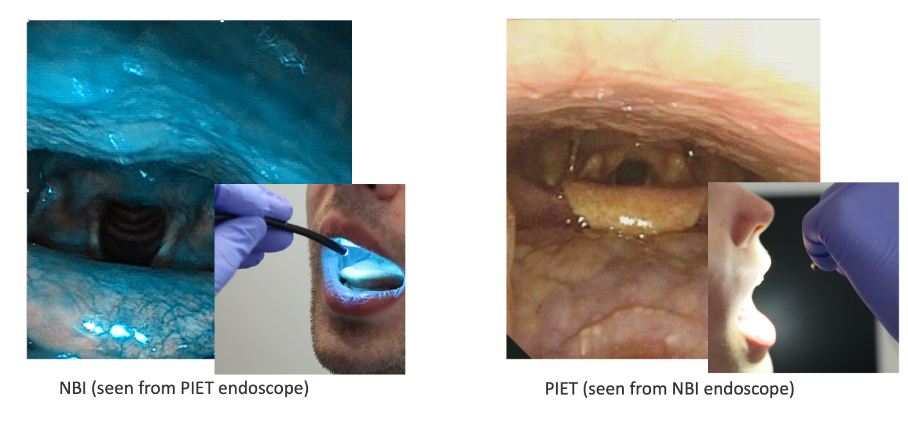


Figure S4 This is what NBI and PIET look like when viewed with the respective other endoscope in the larynx or a camera from outside. NBI light is blue; it increases yellow – when turning NBI on, you sometimes see a short green intermediate step when switching, probably for the red filter. PIET does the whole thing with software - no difference in color (images courtesy of the author).

**Table S1- Raw data**

| Occupation | Task in FEES | Prof. experience (years) | VAS FEES | VAS NBI | VAS PIET | Black 1:10 | Blue 1:10 | Yellow 1:10 | Violet 1:10 | White 1:10 | Orange 1:10 | Green 1:10 | Red 1:10 |
| --- | --- | --- | --- | --- | --- | --- | --- | --- | --- | --- | --- | --- | --- |
| MD | Performing | 31 | 10 | 10 | 3 | P/P/N | P/P/P | E/E/N | N/N/N | P/P/P | E/N/N | N/N/N | E/P/N |
| MD | Performing | 17 | 9,3 | 9,4 | 0,8 | P/P/P | P/P/P | E/E/E | E/E/E | P/P/P | E/N/N | E/N/E | P/E/E |
| MD | Performing | 15 | 10 | 5,8 | 0 | P/P/N | P/P/P | P/P/N | E/E/E | P/P/P | N/N/N | N/N/N | P/P/P |
| MD | Performing | 3 | 6,1 | 5,9 | 0 | P/E/E | P/P/P | N/N/N | P/E/E | P/P/P | N/N/N | P/P/P | P/P/P |
| SLP | Supporting | 15 | 7,7 | 6,4 | 0 | E/N/N | P/P/P | E/E/E | P/E/E | P/P/P | P/N/N | P/E/E | P/E/E |
| SLP | Supporting | 7 | 6,3 | 6,3 | 0 | P/P/P | P/P/P | E/E/E | E/E/E | P/P/P | N/N/N | N/N/N | E/E/E |
| SLP | Supporting | 8 | 4,9 | 6,8 | 0 | E/P/N | E/P/E | N/P/N | P/P/P | P/P/P | P/N/N | P/N/N | P/P/E |
| SLP | Supporting | 30 | 9,2 | 7,8 | 0 | P/P/P | P/P/P | N/N/N | P/P/P | P/P/P | E/E/E | E/E/E | P/P/P |
| SLP | Supporting | 4 | 7,7 | 6,8 | 1,5 | P/P/P | P/P/P | N/P/N | P/E/E | P/P/P | N/N/N | P/N/N | P/P/P |
| Eng. | Technical | 25 | 6,9 | 9,2 | 1 | P/P/P | P/P/P | E/E/E | E/P/P | P/P/P | N/N/N | E/P/P | P/P/P |
| RN | Assisting | 39 | 7 | 7,6 | 0 | P/P/P | P/P/P | N/N/N | P/P/P | P/P/P | P/P/N | N/N/N | P/P/P |
| RN | Assisting | 20 | 7,2 | 7,1 | 0 | P/P/P | P/P/P | E/E/E | E/E/E | P/P/P | N/N/N | E/E/E | P/P/P |
| MD | None | 34 | 0 | 0 | 0 | P/P/P | P/P/P | E/E/E | P/E/E | P/P/P | P/N/N | P/P/N | P/P/P |
| Student | None | 0 | 0 | 0 | 0 | P/P/P | P/P/P | N/N/N | E/N/P | E/N/E | N/N/N | N/N/N | E/P/N |

Table S1 Raw data - rating results of all participants. P=PIET, N=NBI, E=equal. The first value is overall impression, the second is color intensity, and the third is contrast to mucosa. MD=Medical Doctor, SLP=Speech-Language-Pathologist, Eng=Engineer, RN=Registered Nurse, VAS=Visual Analogue Scale.

**Table S2 – used colors** **and their European and US designation, contents, and filtered appearance**

| Color | Appearance WL | Appearance filtered | Consists of | US designation | E-Numbers |
| --- | --- | --- | --- | --- | --- |
| Betanin | Red | Turquoise / Blue | Extract of Beet | N/A | E162 |
| Black | Black | Black | Amaranth Sunset Yellow FCF Brilliant Blue FCF Tartrazine | Food red 9 | E123/ |
|  |  |  |  | Yellow 6 | E110/ |
|  |  |  |  | Blue 1 | E133/ |
|  |  |  |  | Yellow 5 | E102 |
| Blue | Blue | Turquoise / Blue | Brilliant Blue FCF Sunset Yellow FCF Amaranth | Blue 1 | E133/ |
|  |  |  |  | Yellow 6 | E110/ |
|  |  |  |  | Food red 9 | E123 |
| Brilliant Blue | Blue | Turquoise / Blue | Brilliant Blue FCF | Blue 1 | E133 |
| Brown | Brown | Blackish red | Sunset Yellow FCF Cochineal Red A Brilliant blue FCF | Yellow 6 | E110/ |
|  |  |  |  | Ponceau 4R | E124/ |
|  |  |  |  | Blue 1 | E133 |
| Carbon Black | Black | Black | Activated Carbon | N/A (banned) ^21^ | E153 |
| Curcumin (turmeric) | Yellow | Bright red | Curcumin | Natural yellow 3 | E100 |
| Green | Green | Bright red | Brilliant Blue VCF Tartrazine | Blue 1 | E133/ |
|  |  |  |  | Yellow 5 | E102 |
| Cherry Red ("Kirschrot") | Red | Turquoise / Blue | Amaranth Cochineal Red A | Food red 9 | E123/ |
|  |  |  |  | Ponceau 4R | E124 |
| Light Green („Hellgrün“) | Green | Bright red | Chinolin Yellow Indigo Carmine | Food Yellow 13 | E104/ |
|  |  |  |  | Food blue1 | E132 |
| Orange | Orange | Blackish red | Sunset Yellow FCF Tartrazine | Yellow 6 | E110/ |
|  |  |  |  | Yellow 5 | E102 |
| Patent Blue V | Blue | Turquoise / Blue | Patent Blue V | Food Blue 5 | E131 |
| Pink | Pink | Turquoise / Blue | Erythrosin Amaranth | Red No. 3 | E127/ |
|  |  |  |  | Food red 9 | E123 |
| Red | Red | Turquoise / Blue | Cochineal Red A Sunset Yellow FCF | Ponceau 4R | E124/ |
|  |  |  |  | Yellow 6 | E110 |
| Red dye paste | Red | Turquoise / Blue | Carmine | Natural red 4 | E120/ |
|  |  |  | Iron Oxide | N/A | E172 |
| Red dye powder | Red | Turquoise / Blue | Riboflavin Carmine | Vitamin B2 | E101/ |
|  |  |  |  | Natural red 4 | E120 |
| Color continued | Appearance WL continued | Appearance filtered continued | Consists of  continued | US designation  continued | E-Numbers  continued |
| Strawberry Red („Erdbeerrot“) | Red | Blackish red | Cochineal Red A Chinolin Yellow | Ponceau 4R | E124/ |
|  |  |  |  | Food Yellow 13 | E104 |
| Violet | Violet | Turquoise / Blue | Amaranth | Food red 9 | E123/ |
|  |  |  | Brilliant Blue FCF | Blue 1 | E133 |
| White | White | White | Titanium Dioxide | Pigment White 6 | E171 (banned) ^11^ |
| Yellow | Yellow | Bright red | Tartrazine | Yellow 5 | E102/ |
|  |  |  | Sunset Yellow FCF | Yellow 6 | E110 |

Sources: Colors without extra names: Lianyungang Xinai via Amazon, Strawberry Red, Brilliant Blue, Cherry Red, and Light Green: Schreiber Essenzen, Germany. Red dye Powder: Brauns Heitmann, Germany. Red dye paste: Decocino, Germany. Carbon Black: Dr. Oetker, Germany. Patent Blue: Ruf, Germany.
